# Polymorphisms of methylenetetrahydrofolate reductase gene as the genetic predispositions of coronary artery diseases in eastern India

**DOI:** 10.4103/0975-3583.70922

**Published:** 2010

**Authors:** Soujatya Dhar, Sumana Chatterjee, Saumitra Ray, Anjanlal Dutta, Bani Sengupta, Shila Chakrabarti

**Affiliations:** *Thalassemia Research Unit, Vivekananda Institute of Medical Sciences 99, Sarat Bose Road, Kolkata – 700 026, West Bengal, India*; 1 *Departments of Medicine and Cardiology, Ramakrishna Mission Seva Pratishthan, Vivekananda Institute of Medical Sciences 99, Sarat Bose Road, Kolkata – 700 026, West Bengal, India*

**Keywords:** Allele, case–control, methylenetetrahydrofolate reductase, multiple logistic regression, polymorphism

## Abstract

**Background:**

Gene–environment interaction is an important aspect in the development of coronary artery disease (CAD). The mutation (677C-T) of methylenetetrahydrofolate reductase (MTHFR) gene results in a decrease of the enzyme activity that leads to mild hyperhomocysteinemia. Elevated plasma level of homocysteine has been recognized as an independent risk factor for cardiovascular disease. A case–control study was designed to assess whether the prevalence of some MTHFR gene polymorphisms have any role in the development of CAD.

**Materials and Methods:**

The study included unrelated 217 cases with CAD and 255 healthy controls. DNA was extracted from peripheral blood. MTHFR genotypes were identified by seeing the presence or absence of 677C→T mutation obtained by PCR followed by Hinf1 restriction digestion. Multiple logistic regression analysis was carried out to find association between studied genotypes and lifestyle as well as biochemical risk factors.

**Results:**

The T allele was found to be associated with the disease. Significant associations were found with smoking, hypertension, diabetes, and family history of CAD.

**Conclusion:**

The results indicate that MTHFR 677C-T polymorphism has significant association with CADs in the population of eastern India.

## INTRODUCTION

It has been predicted that cardiovascular diseases will increase rapidly in India, and this country will be the host to more than half the cases of heart disease in the world within the next 15 years.[1] Global Burden of Disease Study estimated that India faces the greatest burden due to coronary artery disease (CAD).[2] Ischemic heart disease (IHD), myocardial ischemia (MI), is a disease resulting reduced blood supply to the heart muscle, usually due to CAD (atherosclerosis of the coronary arteries). According to the Framingham Heart Study[3] in USA, played vital role in defining the risk factors for coronary heart disease (CHD) incidence in general population. CAD is a multifactorial disorder that is thought to result from an interaction between genetic background and environmental factors. Major risk factors are lifestyle habits, smoking, alcohol intake, hypercholesterolemia (a condition with high levels of bad cholesterol or low density lipoprotein), diabetes, hypertension (a condition with a blood pressure greater than or equal to 140 mmHg systolic pressure or greater than or equal to 90 mmHg diastolic pressure in an adult), obesity, positive family history, small dense low density lipoprotein (LDL) particles, lipoprotein A, serum homocysteine, and abnormalities in several coagulation factors. Interactions between genetic and environmental factors influence progression of pathological processes, clinical characteristics of disease, and susceptibility to therapeutic treatment.[4] Genetic factors help in explaining the molecular basis of the disorder and in designing prevention and treatment of the disease.

Homocysteine is an emerging new risk factor for cardiovascular disease. Systematic reviews of observational (cohort and case–control) studies have consistently shown a strong, positive, and dose-related association between the serum concentration of total homocysteine (tHcy) and the risk of stroke, which is independent of other vascular factors[5,6] The term homocyst(e)ine is used to define the combined pool of homocysteine, homocystine, mixed disulfides involving homocysteine, and homocysteine thiolactone, and proteinbound homocysteine.[7–9] Protein-bound homocysteine accounts for 70–80% of the total pool. Homocysteine is a nonprotein forming, sulfhydryl-containing amino acid, which results from methionine metabolism. Methionine, an essential amino acid (present in meat, milk, eggs, legumes, etc.), is activated to form *S*-adenosylmethionine (SAM), the universal methyl group donor. Homocysteine acts at the intersection of two metabolic pathways, the transsulfuration pathway and the remethylation cycle. Homocysteine is either transsulfurated to cystathionine or remethylated to methionine depending on methionine supplied by diet.[10] Methylenetetrahydrofolate reductase (MTHFR) is an enzyme which plays an important role in homocysteine metabolism by catalyzing the reduction of 5,10-methylenetetrahydrofolate to 5-methyltetrahydrofolate. 5-Methyltetrahydrofolate is the major circulatory form of folate and carbon donor for remethylation of homocysteine to methionine.[4] The human MTHFR gene is located at chromosome 1p36.3 and consists of 11 exons with a length of 1980 bp.[11] The C to T missense mutation in exon 4 at codon 677 of the MTHFR gene (677C→T), which causes an alanine (A) to valine (V) substitution in the MTHFR protein, produces a thermolabile form of the enzyme, reduces enzyme activity, and results in increased plasma homocysteine.[12] Elevated plasma level of homocysteine has been recognized as an independent risk factor for cardiovascular disease,[13] but the strength of the relationship and the interaction of plasma homocysteine with other risk factors are unclear.[14,15] The main mechanisms of HCY atherogenic action are thought to be LDL oxidation, inhibition of vascular endothelium growth combined with stimulation of smooth muscular cells proliferation, and interference with the coagulation and fibrinolytic systems.[14,16] A point mutation in the gene encoding MTHFR has been associated with elevations in homocysteine levels in homozygous carriers (TT genotype) and is considered as an independent risk factor for vascular diseases.[17] Homozygosity for this 677T variant was shown to be associated with increased plasma Hcy levels, particularly when folate status is low[18,19] and it has been described as a risk factor for CAD, although this association is not clearly established at present.[20]

To the best of our knowledge, this is the first study to report on probable association of certain MTHFR polymorphisms as predisposing factors in the development of the CAD and their interactions with relevant biochemical and lifestyle factors in the population of eastern India.

## MATERIALS AND METHODS

### Choice of study subjects

#### Choice of individuals

A total of 217 clinically diagnosed cases with CADs were chosen from the outdoor of Cardiology Department of Ramakrishna Mission Seva Pratishthan. A total of 255 age- and sex-matched healthy controls were taken for the study with no heart-related complains. All subjects underwent coronary angiography to confirm the presence of the disease.

#### Collection of personal and family history

Detailed history regarding their clinical, lifestyle, and socioeconomic conditions were collected. Detailed status of the relevant biochemical parameters was noted from their recent investigations.

#### Maintenance of ethical background

Informed written consents were obtained from all subjects. All the study subjects were Bengali, Indian and belonged to the same ethnic group. The research protocol was approved by the ethical and research advisory committee of the institution.

### MTHFR polymorphism study

#### Collection of blood

Blood samples, 2–3 mL, were collected from each individual in an EDTA vial.

#### Isolation of DNA

Genomic DNA was extracted from peripheral blood leukocytes by salting out method.[21]

#### PCR–RFLP method to detect the genotypes

MTHFR genotype study was carried out to identify the presence of 677C→T mutation as described by Frosst *et al*.[12] The PCR product (198 bp) was digested with Hinf1 restriction enzyme.

#### Agarose gel electrophoresis to detect the genotypes

The mutation 677C-T creates a Hinf1 recognition sequence, and the product was digested into 175 bp and 23 bp fragments. The products of restriction digestion were separated on a 3% agarose gel and visualized by ethidium bromide staining. Genotypes of all individuals were noted as CC: 198 bp; CT: 198 bp, 175 bp, 23 bp; and TT: 175 bp, 23 bp.

All the results were checked twice in double blind.

### Statistical analysis

To test for significance of observed interactions, likelihood ratio tests were conducted using a Chi-square test. The odd-ratios (OR) for different association models were calculated with 95% confidence interval (CI) and simultaneously *P* values were calculated. A value of *P* < 0.05 was considered as statistically significant. Extent of other risk factors was compared between cases and controls and across genotype levels. The stepwise logistic regression analysis was performed with the SPSS Statistical Package (version 14) for the determination of the independent risk factors for CAD. Multivariate logistic regression was used to obtain the OR estimate and 95% CI for the main effects of smoking and main enzyme genotype. An adjustment was made for the potential confounding effects of age, sex, alcohol consumption, tobacco consumption, family history, coexistence of hypertension, diabetes, etc. Dummy variables were created each representing the combination of category and genotype. In addition, interaction terms including duration or amount of exposure were evaluated for statistical significance.

## RESULTS

Different lifestyle as well as demographic characteristics of the subjects are shown in Table 1. Out of all cases, 44.7% were both cigarette and bidi (a dry-rolled temburni leaf containing fine tobacco dust) smokers and 78.06% of the controls were addicted to other tobacco habits (catechu, zarda, etc). Though the frequency of alcohol intake was higher among the cases than the control, it did not have any statistical impact on the disease. Observed data strongly correlate the incidence of the disease with family history. Statistical significance found in the case of diabetes and hypertension, which elevates the risk of the disease.

**Table 1:** Characteristics of the study subjects

	Cases (*N* = 217)	P*	Controls (*N* = 255)
Age (years)			
Range	29–81	–	18-66
Average	42.60		40.53
Gender (n,%)			
Male	119 (54.8)	<0.05	140 (54.90)
Female	98 (45.16)		115 (45.09)
Lifestyle habits (n,%)			
Smoking	97 (44.7)	<0.01	49 (19.21)
Other tobacco habit	129 (59.44)	<0.01	199 (78.03)
Alcohol intake	89 (41.01)	NS	23 (9.01)
Family history (n, %)	72 (33.33)	<0.001	36 (14.11)
Diabetes (n, %)**	82 (37.78)	<0.001	32 (12.54)
Hypertension (n, %)	183 (84.33)	<0.01	23 (9.01)***

* P = Calculated as Chi-square test.

** Fasting blood glucose ≥ 120 mg/dL.

*** Systolic pressure > 130 mmHg. Diastolic pressure > 80 mmHg. NS = Nonsignificant.

Biochemical parameters such as HDL, LDL, serum cholesterol, and serum triglyceride were shown in Table 2. These parameters were significantly associated with CAD. Increased level of total cholesterol and LDL cholesterol were obtained in the cases as expected. Serum LDL cholesterol and triglyceride concentration were elevated in CAD patients in comparison with control subjects. The HDL cholesterol levels were significantly lower in CAD patients than in controls (*P* < 0.02).

**Table 2:** Association of biochemical risk markers among the study subjects

	Cases (N=217)	P*	Control (N = 255)
HDL (mg/dL)	49.6 ± 2.4	<0.02	66.2 ± 2.4
LDL (mg/dL)	143 ± 2.4	<0.02	115 ± 3.4
Serum cholesterol (mg/dL)	188 ± 5.2	<0.04	173.5 ± 3.5
Serum triglyceride (mg/dL)	157 ± 3.2	<0.02	132.2 ± 2.4

HDL = High density lipoprotein; LDL = Low density lipoprotein.

* P = Calculated as Chi-square test.

Distribution of different MTHFR genotypes and the allele frequencies of the study groups were shown [Tables 3 and 4], where significant differences were found between cases and controls. The frequencies were almost compatible with Hardy–Weinberg equilibrium. We found that the prevalence of CC genotype was the highest in subjects. The prevalence of C allele was significantly high in subjects.

**Table 3:** Distribution of MTHFR genotypes and allele frequencies of the study groups

	Genotype frequencies			Allele frequencies	
	CC	CT	TT	C allele	T allele
Case (*n*)	0.512	0.21	0.26	0.63	0.40
					*H* = 1.01*
Control (*n*)	0.73	0.14	0.12	0.8	0.2
					*H* = 1*

* Chi-square tests (with df = 2) were performed to check whether the population were in Hardy–Weinberg equilibrium.

**Table 4:** Distribution of MTHFR genotypes in the study subjects (overall)

Genotype	Cases (n, %)	Control(n, %)	Crude OR(CI%)	P*
CT	47 (21.6)	36 (14.11)	2.17 (88.31–102.19)	<0.05
TT	58 (26.72)	33 (12.9)	2.92 (90.52–103.98)	<0.05
CC	112 (51.6)	186 (72.94)	–	–
T allele	163 (38.03)	102 (36.12)	3 (228.82–243.18)	<0.10
C allele	271 (61.96)	408 (63.87)	–	–

OR = Odd ratio; CI = confidence interval; NS = nonsignificant.

* P values obtained through Chi-square analysis of the differential genotypes.

Interactions among different polymorphisms of MTHFR gene and age, sex, diabetes, hypertension, positive family history, different forms of tobacco use and alcohol intake were summarized with a view to increase the risk of CADs [Table 5]. It was found that smoking (>35 shots/week) and tobacco intake (2–3 shots/day), diabetes, hypertension, and family history of CAD were significantly associated with TT and CT genotypes. Stepwise logistic regression analysis showed that the T allele of MTHFR gene was an independent risk factor of CADs (OR: 3; CI: 228.82– 243.18; *P* < 0.10).

**Table 5:** Association of MTHFR genotypes with other risk factors

	TT				CT				CC	
	Case (n,%)	Control (n,%)	OR,95%CI	P*	Case (n,%)	Control (n,%)	OR, 95%CI	P*	Case	Control
Sex										
Male	31(14.3)	20(7.8)	0.656 (0.383–1.12)	0.123	22(10.13)	17(6.7)	0.425 (0.238–0.757)	0.004	59(27.19)	97(38.03)
Female	27(12.4)	13(5.09)			25(11.52)	19(7.4)			53(24.42)	89(34.9)
Age										
< 45 years	27(12.44)	14(5.5)	1.381 (0.789 –2.44)	0.266	22(10.13)	20(7.8)	1.195 (0.641–2.22)	0.575	48(22.11)	77(30.2)
>45 years	31(14.28)	19(7.4)			25(11.5)	16(6.3)			64(29.5)	109(42.74)
Smoking										
Never	9(4.14)	11(4.3)			12(5.5)	15(5.8)			42(19.35)	74(29.01)
Exsmokers	14(6.5)	6(2.3)	0.26 (0.117–0.579)	0.001	8(3.6)	4(1.6)	0.214 (0.093–0.4796)	0.002	22(10.13)	48(18.82)
<35 shots/week	16(7.4)	9(3.5)	0.243 (0.08 – 0.73)	0.012	17(7.8)	9(3.5)	0.25 (0.084 – 0.762)	0.015	20(9.21)	31(12.15)
>35 shots/week	19(8.7)	7(2.7)	1.42 (0.144 – 1.28)	0.003	10(4.6)	8(3.13)	1.34 (1.109 – 1.113)	0.005	28(12.90)	33(12.94)
Other tobacco intake									
2-3 shots/day	22(10.18)	13(5.09)	1.2 (0.124 – 0.447)	0.002	25(11.5)	16(6.27)	1.2 (0.121–0.455)	0.001	62(28.57)	83(32.5)
Never	36(16.6)	20(7.8)			22(10.1)	20(7.8)			50(23.04)	103(40.39)
Alcohol intake										
Heavy	19(8.8)	6(2.4)	0.30 (0.072 – 1.247)	0.098	11(5.06)	8(3.14)	0.310 (0.069–1.395)	0.127	20(9.21)	25(9.8)
Occasional	17(7.8)	4(1.5)	0.129 (0.054 – 0.310	0.08	15(6.91)	10(3.9)	0.126 (0.053 – 0.301)	0.07	28(12.9)	45(17.6)
Never	22(10.13)	23(9.0)			21(9.7)	18(7.05)			64(29.49)	116(45.49)
Coexistence of diabetes									
Yes	38(17.51)	12(4.7)	1.164 (1.07 – 1.34)	0.02	19(18.75)	13(5.09)	1.28 (1.12 – 1.64)	0.003	38(17.59)	67(26.27)
Never	20(9.21)	21(8.23)			28(12.9)	23(9.01)			74(34.10)	119(46.7)
Coexistence of high LDLC									
Yes	36(16.6)	13(5.09)	0.254 (0.130–0.499)	0.00	26(11.98)	16(6.27)	0.472 (0.248–0.899)	0.222	47(21.65)	73(28.62)
Never	22(10.13)	20(7.84)			21(9.67)	20(7.84)			63(29.03)	113(44.31)
Family history of CAD									
Yes	32(14.74)	11(4.31)	1.34 (1.167–1.7)	0.004	27(12.44)	17(6.66)	1.2 (1.117–1.27)	0.003	27(12.44)	52(20.39)
No	26(11.98)	22(8.62)			20(9.21)	19 (7.45)			85(39.17)	134(52.54)
Coexistence Of hypertension									
Yes	48(22.11)	7(2.74)	1.015 (1.006 – 1.035)	0.005	29(13.36)	7(2.74)	1.02 (1.008 – 1.049)	0.008	87(40.09)	32(12.54)
Never	10(4.6)	26(10.19)			18(8.29)	29(11.37)			25(11.52)	154(60.39)

OR: Odd Ratio; CI: Confidence Interval.

* calculated by χ^2^ analysis comparing genotype distributions among case and control subjects.

## DISCUSSION

Several studies have showed the positive and negative associations of different MTHFR gene polymorphisms and various forms of CVDs. Risk factors and as well as genetic factors also play an important role in the development of CAD. As per smoking and alcohol intake is concerned, our results partially support some of the previous works.[22] We observed significant association of extensive smoking and other tobacco habits with the disease. However, alcohol intake did not have any role on the disease development. Further analysis showed significant association between smoking (>35 shots/week), other tobacco habits with CT and TT genotypes but no association was found in the case of alcohol intake.

Some authors suggested that the 677C-T mutation in the MTHFR gene is a potential risk factor for cardiovascular diseases. Frosst *et al*, reported that C677T mutation in the heterozygous or homozygous state is a potential risk factor for cardiovascular events. Gulec *et al*,[23] found a relationship between the C677C-T transition in the MTHFR gene and myocardial infarction in Turkish men. Evidence of MTHFR C677T polymorphism may slightly predispose to young adults to the development of stroke on German population.[24] A study investigated that MTHFR 677CT polymorphism, though associated with homocysteine levels, confers no significant risk of CAD in the Pakistani population.[25] Limited data are available for other Asian populations, especially Indians.[26–28] Nair *et al*,[26] reported that heterozygosity for thermolabile MTHFR mutation was associated with hyperhomocysteinemia, which could be a risk factor for CAD in the Indian population. A study reported that no such significant differences between CAD patients and controls in the frequency of alleles and genotypes of the MTHFR gene in north Indian CAD patients.[29] A study on south Indian population reported that T-allele frequency was almost similar between the newborns and adults; however, a higher T-allele frequency was observed in females than males.[30] Another study on Indian population reported that no significant difference in frequencies of MTHFR C677T genotypes was found between stroke patients and the control group.[31] The aim of our study was to look for the polymorphisms of MTHFR in the development of CAD in the general population of eastern India. We found significant difference between CAD patients and controls in the frequency of alleles and genotypes of MTHFR. Coexistence of diabetes and hypertension has been reported to be a risk factor for the development of CAD.[32] Both hypertension and diabetes were more significantly associated with CT and TT genotypes in our study. Family history of CHD has been well recognized in some studies.[33,34] However in terms of risk factor associated with CAD, we have also observed significantly higher prevalence of CT and TT genotypes among the cases who had strong history of development of CAD in their first degree relatives than the controls. The LDL, HDL, serum cholesterol, and serum triglyceride were found to have statistical correlations with the diseases at different significant levels. Few agreed that high LDL cholesterol and low HDL cholesterol are independent risk factors for CVD[29,35] and our study also supports it.

In spite of several precautions, our study has certain limitations. Our study was hospital-based and could result in biased selection in place of random sampling. It has been suggested that studies with hospital controls can provide lower risk estimates, since diseases of controls could be associated with the polymorphisms under study. On the other hand, 217 individuals in no way represent a population status. Therefore, the study is to be continued with more number of study cases. Thus, the associations and the underlying mechanisms of lifestyle factors with the metabolic enzymes gene polymorphisms still need further studies with large-scale (population-based) samples and modified designs.
